# The association between long-term exposure to low-level PM_2.5_ and mortality in the state of Queensland, Australia: A modelling study with the difference-in-differences approach

**DOI:** 10.1371/journal.pmed.1003141

**Published:** 2020-06-18

**Authors:** Wenhua Yu, Yuming Guo, Liuhua Shi, Shanshan Li

**Affiliations:** 1 Department of Epidemiology, School of Public Health and Management, Binzhou Medical University, Yantai, Shandong, China; 2 Department of Epidemiology and Preventive Medicine, School of Public Health and Preventive Medicine, Monash University, Melbourne, Australia; 3 Gangarosa Department of Environmental Health, Rollins School of Public Health, Emory University, Atlanta, Georgia, United States of America; U.S. EPA, UNITED STATES

## Abstract

**Background:**

To date, few studies have investigated the causal relationship between mortality and long-term exposure to a low level of fine particulate matter (PM_2.5_) concentrations.

**Methods and findings:**

We studied 242,320 registered deaths in Queensland between January 1, 1998, and December 31, 2013, with satellite-retrieved annual average PM_2.5_ concentrations to each postcode. A variant of difference-in-differences (DID) approach was used to investigate the association of long-term PM_2.5_ exposure with total mortality and cause-specific (cardiovascular, respiratory, and non-accidental) mortality. We observed 217,510 non-accidental deaths, 133,661 cardiovascular deaths, and 30,748 respiratory deaths in Queensland during the study period. The annual average PM_2.5_ concentrations ranged from 1.6 to 9.0 μg/m^3^, which were well below the current World Health Organization (WHO) annual standard (10 μg/m^3^). Long-term exposure to PM_2.5_ was associated with increased total mortality and cause-specific mortality. For each 1 μg/m^3^ increase in annual PM_2.5_, we found a 2.02% (95% CI 1.41%–2.63%; *p* < 0.01) increase in total mortality. Higher effect estimates were observed in Brisbane than those in Queensland for all types of mortality. A major limitation of our study is that the DID design is under the assumption that no predictors other than seasonal temperature exhibit different spatial-temporal variations in relation to PM_2.5_ exposure. However, if this assumption is violated (e.g., socioeconomic status [SES] and outdoor physical activities), the DID design is still subject to confounding.

**Conclusions:**

Long-term exposure to PM_2.5_ was associated with total, non-accidental, cardiovascular, and respiratory mortality in Queensland, Australia, where PM_2.5_ levels were measured well below the WHO air quality standard.

## Abstract 2

Wenhua Yu and colleagues reveal how long term PM2.5 exposure may be associated with higher risk of cardiac or respiratory disease mortality.

## Author summary

**Why was this study done?:**

- Long-term exposure to fine particulate matter (PM_2.5_) is identified as one of the leading risk factors for death.
  However, previous studies have been subject to uncertainty and potential biases due to unmeasured or omitted confounders.
- The difference-in-differences (DID) approach could mimic an experimental research design using observational study data to provide an effect estimate by adjusting for unmeasured confounders.
- Few studies investigate the influence of long-term exposure to low-level PM_2.5_ on cause-specific mortality, particularly for the concentrations consistently well below the current WHO annual standard.

**What did the researchers do and find?:**

- We used a variant of the DID approach to investigate the association between long-term exposure to low-level PM_2.5_ and cause-specific mortality in Queensland, Australia, between 1998 and 2013.
- We studied 242,320 registered deaths from 7 categories of diseases in Queensland and found a 2.02% increase in total mortality for each 1 μg/m^3^ increase in annual PM_2.5_.
- The associations were consistent across cardiovascular, respiratory, and non-accidental mortality.

**What do these findings mean?:**

- Our findings support growing evidence that, when controlling for unmeasured confounders by design, low-level PM_2.5_ exposure may contribute to increased risk of mortality.
- Long-term exposure to PM_2.5_-mortality association even at PM_2.5_ levels below the WHO air quality guideline informs decision-making for environmental health management and making air pollution control strategies.
- The findings should be interpreted cautiously because the DID design is based on the assumption that all potential spatial-temporal factors were controlled, and the variability of confounders was held constant during the study period.

## Introduction

Ambient particulate matter with diameters less than 2.5 micrometers (fine particulate matter; PM_2.5_) has been identified as the fifth leading mortality risk factor in 2015, contributing to 4.2 million deaths [1]. While a growing body of literature [2–6] has examined the causal effect of PM_2.5_ on mortality, scientific evidence remains weak. Causal model approaches seek to mimic randomized controlled trials, whereby exposure is measured independent of the other predictors of the health outcome. This effectively eliminates the possibility of confounding. Propensity score matching and inverse probability weighting are the most common approaches to reweight the study populations to ensure exposure is independent of all measured confounders [7]. Wu and colleagues applied a propensity score approach to estimate the causal effect of long-term PM_2.5_ exposures on mortality in New England [8]. Wang and colleagues employed a doubly robust causal modelling approach with inverse probability weights to estimate the hazards of long-term exposure to PM_2.5_ on survival in the southeast United States [9]. However, this method only accounts for measured confounders and unmeasured biases that are highly correlated with the measured confounders [10]. Another approach to estimating the causal effect is the regression discontinuity design. This approach compares observations lying closely on either side of a threshold to estimate the average treatment effect [11]. In one such study, Avraham Ebenstein and colleagues employed the regression discontinuity design to investigate the causal effect of particulate matter with a diameter of less than 10 micrometers (PM_10_) on Chinese life expectancy by assigning different coal subsidy policies for indoor heating on both sides of Huai River [12]. However, the estimated effects could still be misled by other potential confounders that occur at the same threshold [13].

To address this limitation, a difference-in-differences (DID) approach has been proposed [14]. DID estimates the effect of an exposure or treatment on an outcome by comparing the average change over time in the outcome variable for a treatment group, compared to the average change over time for a control group. A DID method mimics an experimental research design using observational study data to provide a causal estimate by adjusting for unmeasured confounders. It assumes that differences between outcomes that change over time are caused by the differences between the observed and counterfactual exposures, rather than by other factors such as socioeconomic status (SES), population, smoking, and obesity, because such factors among other unmeasured confounders are similar between locations across time. Several studies have utilized the DID method to explore the association between PM and mortality [2,15–18]. For example, Corrigan and colleagues used a DID approach to examine the association between changes in PM_2.5_ and changes in cardiovascular mortality rates before and after the implementation of a new PM_2.5_ National Ambient Air Quality Standards (NAAQS) in the US [17]. Wang and colleagues estimated the causal effect of long-term exposure to PM_2.5_ on mortality in New Jersey by developing a variant of the DID approach [2]. Similar models were used to assess the long-term PM_2.5_-mortality association in 207 cities across the US [18].

Most of the DID studies estimated the effect of long-term exposure to PM and mortality at relatively high concentrations [15,16,19]. For example, Matteo Renzi and colleagues used a DID approach to estimate the effect of annual PM_10_ exposure with a range of 21.9 ±4.9 μg/m^3^ in the Latium region, Italy [15]. Another study’s estimated mortality changes were specifically attributable to high exposure to PM_2.5_ in China between 2000 and 2010 [19]. However, growing studies have shown that there is no obvious PM_2.5_ threshold for the PM_2.5_-mortality association even at PM_2.5_ levels under the World Health Organization (WHO) air quality guideline (10 μg/m^3^ of annual average PM_2.5_). A meta-analysis including 14 studies conducted on participants with average exposure to PM_2.5_ below 10 μg/m^3^ supported the nonlinear PM_2.5_-mortality exposure-response association, where the effect increased rapidly at lower concentrations [20]. However, few studies [3,4] have explored the association of PM_2.5_ with mortality below a concentration of 10 μg/m^3^. Therefore, exploring the relationship between long-term low levels of PM_2.5_ and cause-specific mortality is warranted, particularly in areas with consistently low PM_2.5_ concentrations in Australia.

In addition, significant differences exist between rural and urban areas in terms of population characteristics, air pollution concentration, and chemical and physical composition. These may contribute to different health effects on cause-specific mortality, especially in Queensland, where approximately half of the population lives in the Brisbane metropolitan area. To address this gap, we assessed the association of long-term exposure to low-level PM_2.5_ with total mortality and cause-specific mortality from cardiovascular, respiratory, and non-accidental causes in Queensland and Brisbane during 1998–2013 using a DID approach [2].

## Methods

This study is reported as per the Strengthening the Reporting of Observational Studies in Epidemiology (STROBE) guideline (S1 STROBE Checklist). We did not include a formal prospective analysis plan, but the study methodology and analysis were planned before conducting the DID approach, with the exception of the analysis of relative change rate and the effect modification by stratifying the age groups. This study was approved by the Monash University Human Research Ethics Committee.

### Study area

**Fig 1:**
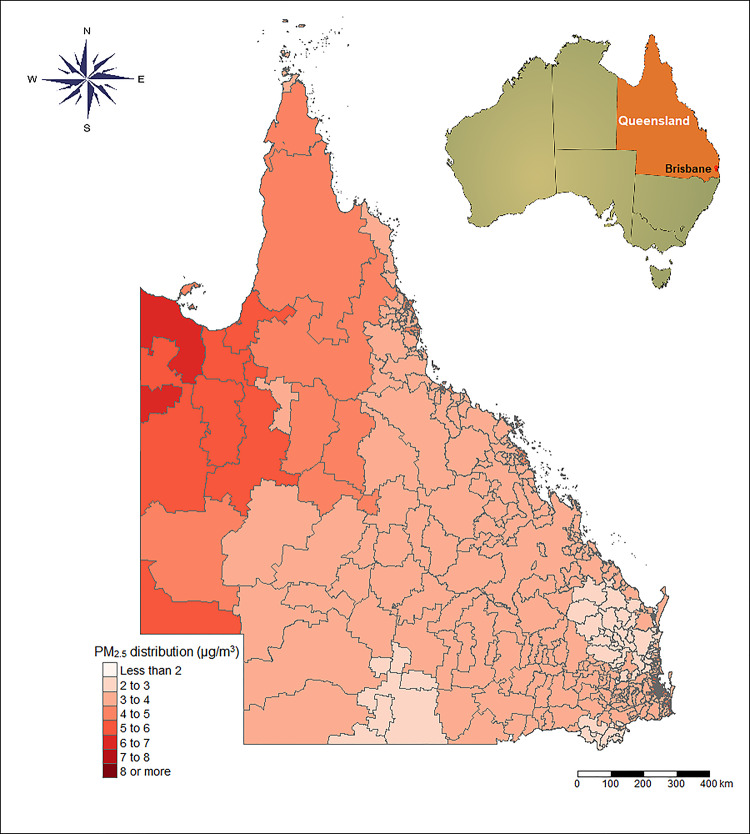
The annual average PM_2.5_ concentrations in Queensland during 1998–2013. PM_2.5_, fine particulate matter (particulate matter with a diameter of <2.5 μm). *The base map was obtained from Australian Statistical Geography Standard (ASGS), https://www.abs.gov.au/websitedbs/d3310114.nsf/home/digital+boundaries, CC BY 2*.*5 AU*.

Queensland is the second largest state in Australia, with an area of 1,852,642 square kilometers, and located in the northeast of the country [21] (Fig 1). Typically, Queensland experiences two weather seasons: a winter with mild temperatures and minimal rainfall, and a humid summer with both high temperatures and high levels of rainfall. As of 2016, it was composed of 4,689,134 inhabitants (449 postcode zones), mainly concentrated along the coast and in the state’s South East. Brisbane was the largest city in the state, with 2,109,466 residents in 2016. In this study, Brisbane was divided into a total of 119 postcode zones.

### Data resources

Individual-level data, such as sex, age, and total and cause-specific mortality from 1 January, 1998, to 31 December 2013, were obtained from Queensland Health (https://www.health.qld.gov.au/public-health). Daily data on temperature were obtained from the Scientific Information for Land Owners (SILO) data set (https://www.longpaddock.qld.gov.au/silo/). Annual population and SES census data (including weekly income per person, Socio-Economic Indexes for Areas [SEIFA]) were provided by the Australian Bureau of Statistics (https://www.abs.gov.au/).

All mortality data were coded with the ICD-9 code before 1999 or ICD-10 code after that time. Specifically, the diseases of interest included the following: mental and behavioral disorders (F00–F99), diseases of the nervous system (G00–G99), diseases of the circulatory system (I00–I99), diseases of the respiratory system (J00–J99), diseases of the digestive system (K00–K93), diseases of the genitourinary system (N00–N99), and external causes of morbidity and mortality (V01–Y98). In this study, we focus on the annual counts of death from non-accidental causes (all above diseases except for V01–Y98), cardiovascular (ICD-9: 390–459, ICD-10: I00–I99), and respiratory causes (ICD-9: 460–519, ICD-10: J00–J99) during the study period (1998–2013).

### Exposure data

The annual mean PM_2.5_ data were derived from the Atmospheric Composition Analysis Group, which estimated annual PM_2.5_ concentrations at 0.01° × 0.01° (approximately 1 km × 1 km) spatial resolution globally using a Geographically Weight Regression with an out-of-sample cross-validated *R*^2^ of 0.81. The regression incorporated satellite data, simulated aerosol composition, and land use information [22]. The population-weighted annual mean PM_2.5_ concentrations were assigned to each postcode area.

### Statistical analyses

#### DID method

This study was based on a variant of DID design [2] to investigate the relationship of PM_2.5_ with total mortality and cause-specific mortality (non-accidental, cardiovascular, and respiratory causes). The substance of the DID design is that through comparing the same population to itself at different time points, some unmeasured individual and behavioral factors that remain constant over time have been controlled. In this study, the DID design was used to estimate the effect of PM_2.5_ on mortality by comparing the concordance between differences in counts of cause-specific deaths and differences in PM_2.5_ over time (from 1998 to 2013) in a given location (449 spatial units of postcode areas in this study). Specifically, a typical DID model is as follows:

$${Y_{ct}}^{A=\alpha }=\beta_{c0}+\beta_{1}\alpha +\beta_{2}Z_{c}+\beta_{3}U_{t}+\beta_{4}W_{ct}+\epsilon$$ (1)

where *Y*_*ct*_*A* ^= *α*^ is the outcome in location *c* and year *t* under exposure *A* = *α*, *α* is PM_2.5_ concentration, *Z*_*c*_ reflects spatial confounders with minimal variability over the time period (e.g., SES); *U*_*t*_ represents temporal confounders that change over time but not among locations; and *W*_*ct*_ denotes confounders that vary across time and locations. Therefore, differences in outcomes between time periods will be

$${Y_{ct}}^{A=\alpha_{t}}-{Y_{c(t-1)}}^{A=\alpha_{t-1}}=\beta_{1}(\alpha_{t}-\alpha_{t-1})+\beta_{3}(U_{t}-U_{t-1})+\beta_{4}{(W}_{c.t}-W_{c.t-1})$$ (2)

whereby *β*_c0_ and *Z*_*c*_ have cancelled out because effects occur simultaneously in the location *c*. If we take the difference of these differences above between locations *c* and *c*^^^, we have

[YctA=αt−Yc(t−1)A=αt−1]−[Yc^tA=bt−Yc^(t−1)A=bt−1]=β1[(αt−αt−1)−(bt−bt−1)]+β4[(Wc.t−Wc.t−1)−(Wc^.t−Wc^.t−1)] (3)

where *b* is the exposure in location *c*^^^. If the changes in *W*_*c*.*t*_ between time *t* − 1 and *t* are the same in both locations, [(*W*_*c*.*t*_−*W*_*c*.*t*−1_)−(*W*_*c*^.*t*_−*W*_*c*^.*t*−1_)] will equal zero. The difference in outcomes between locations relies solely on changes in exposure (i.e., the causal estimate). Hence, the benefit of this approach is that the known and unknown confounders have been adjusted. Historically, the DID model has been used in two places over two study periods; nevertheless, Wang and colleagues [2] developed this approach using a generalized case with multiple locations and time periods.

In our study, we fit a model as follows:

$$\ln\left[E(Y_{c,t})\right]=\beta_{c0}+\beta_{1}{PM}_{c,t}+\beta_{2}I_{c}+\beta_{3}I_{t}+\beta_{4}{Temp}_{sum}{+\beta }_{5}{Temp}_{win}{+\beta }_{6}sd\_temp_{sum}{+\beta }_{7}sd\_temp_{win}{+\beta }_{8}SEIFA+offset\left({log(P}_{c,t})\right)$$ (4)

where *Y*_*c*,*t*_ denotes the number of deaths in spatial unit *c* and year *t*; *PM*_*c*,*t*_ denotes the annual mean concentration of PM_2.5_ in unit *c* and time *t*; *I*_*c*_ is a dummy variable for each spatial unit in 449 postcode areas in Queensland; *I*_*t*_ represents a dummy variable for each year from 1998 to 2013; *Temp*_*sum*_, *Temp*_*win*_ and *sd*_*temp_sum_*, *sd*_*temp_win_* reflect average temperatures and their standard deviations (SDs) for both summer and winter, respectively; and *sd*_*temp_sum_* and *sd*_*temp*_*win*_ were included to control the fluctuations and variations of seasonal temperature [23]. SEIFA was the Socio-Economic Indexes for Areas, which in this model, reflects the level of economic development in a particular place. We added *offset*(log(*P*_*c*,*t*_)) as an offset term using logarithms of the annual population of each place. The outcomes were presented as percent increase risk of cause-specific mortality for 1 μg/m^3^ increase in annual PM_2.5_.

Our model is based on the following assumptions: (1) The DID relies on a parallel trend assumption that in the absence of intervention (e.g., in the absence of the impact of PM_2.5_ or the concentration of PM_2.5_ remains constant in this study), the unobserved differences among locations are constant over time. Although there is no statistical test for this assumption, annual trends were evaluated visually for relative changes in PM_2.5_ and mortality rate over 16 years. We applied a relative change rate (*RC*_*c*,*t*_) to calculate the percentage change for each area unit in each year with the following equation: *RC*_*c*,*t*_ = (*R*_*c*,*t*_−*E*_*c*_)/*E*_*c*_×100; $E_{c}=\frac{\sum_{t=1998}^{2013}R_{c,t}}{16}$, where *RC*_*c*,*t*_ denotes the annual percent changes of PM_2.5_ or mortality rate in area *c*, year *t*; *R*_*c*,*t*_ is the annual concentration of PM_2.5_ or the cause-specific mortality rate in the same stratum; and *E*_*c*_ denotes the average of *R*_*c*,*t*_ from 1998 to 2013 in each area unit. (2) We assumed that, apart from seasonal temperature, no predictors exhibit differential spatial-temporal variations in relation to the exposure [2,15]. Under that assumption, overall spatial and temporal confounding is removed from the DID design. However, if this assumption is violated, the DID design is still subject to confounding.

#### Conditional Poisson regression

We applied a conditional Poisson regression model [24] to perform the DID design, to estimate the association of long-term exposure to PM_2.5_ with mortality using the “gnm” package in R software (version 3.2.5). We adjusted for summer and winter temperatures and SEIFA effect, conditional on strata of spatial units. Additionally, we also estimated whether the effect was modified by different proportions of age and sex based on Census 2016 data using subgroup analyses.

#### Sensitivity analysis

We performed sensitivity analyses to test the robustness of the results using a random-effects meta-analysis, estimating whether the effect estimate in a specific population was disparate from the pooled effect estimate in Queensland. The potential nonlinearity of the association between PM_2.5_ and total and cause-specific mortality was examined using cubic splines with 3 degrees of freedom. We also modelled the summer and winter temperatures using natural splines with 3 and 4 degrees of freedom, respectively, to assess for modification of health effects of PM_2.5_ by season. In order to control the impact of the population age structure, we tested the effect modification by stratifying the population into two age groups: <65 years and ≥65 years, using the offset term of age-specific person-years. All analyses were conducted using R software (version 3.2.5).

## Results

**Fig 2:**
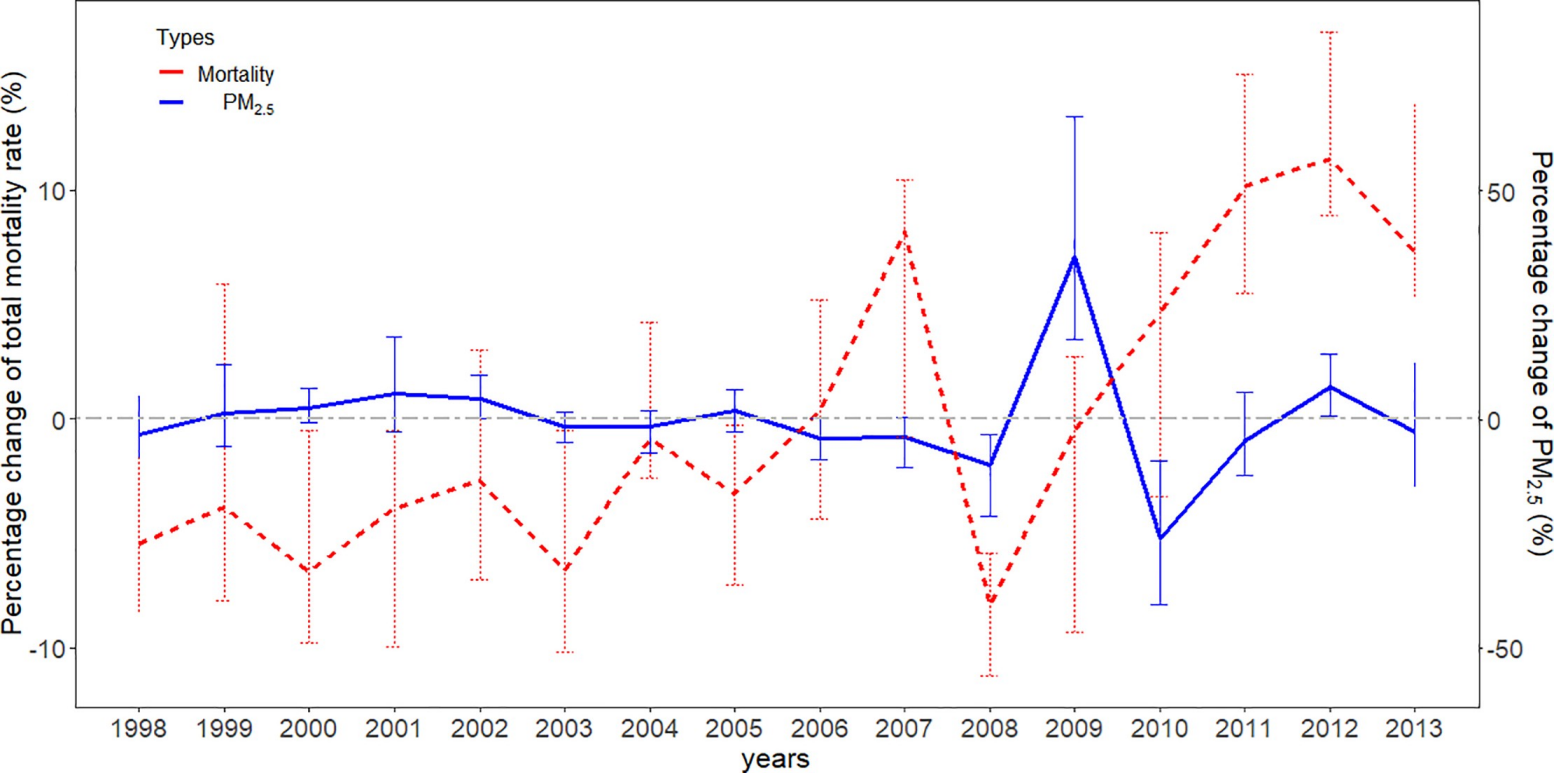
The percentage changes in mortality rate and PM_2.5_ concentrations in area units during 1998–2013. The percentage changes are the percent difference between the values of PM_2.5_ or mortality rate (per 1,000 persons) in area unit specific to each year and the average of the values from 1998 to 2013 in the same area unit, divided by the average of the values in area unit specific to the time from 1998 to 2013. The error bars for both lines are the range (maximum–minimum) of all areas. PM_2.5_, fine particulate matter (particulate matter with a diameter of <2.5 μm).

We studied 242,320 deaths in 7 categories of diseases (ICD10: F00–F99, G00–G99, I00–I99, J00–J99, K00–K93, N00–N99, V01–Y98) from 1998 to 2013, which accounted for 60.5% of registered deaths during the study period. Specifically, 217,510 non-accidental deaths, 133,661 deaths from cardiovascular diseases, and 30,748 deaths from respiratory diseases in the Queensland region were investigated. Table 1 displays the distribution of deaths, PM_2.5_, and temperature in Brisbane and Queensland over the study period. In short, 81.9% of deaths were over 65 years old and 50.8% were male. The average PM_2.5_ concentrations were 6.0 μg/m^3^ (range: 2.13–8.00 μg/m^3^) in Brisbane **(**S1 Fig**)** and 3.63 μg/m^3^ (range: 1.63–9.00 μg/m^3^) in Queensland (Fig 1), with interquartile ranges (IQRs) of 2.00 μg/m^3^ and 2.23 μg/m^3^, respectively. There was a decrease of 9.33% in the standardized death rate in Queensland over the 16-year study period (S1 Table). There was no significant change in PM_2.5_ or temperature during the study period. The annual average PM_2.5_ ranged from 2.01 to 5.28 μg/m^3^, along with a downward trend from 1998 to 2008 and with slight increase between 2008 and 2013. The range of seasonal mean temperature varied from 24.5°C to 26.5°C in summer and from 15.0°C to 16.4°C in winter **(**S1 Table**)**. In terms of the relative changes of PM_2.5_ and mortality rate, we calculated the percentage changes (*RC*_*c*,*t*_) of both the annual concentration of PM_2.5_ and the total mortality rates for all area units in the study period, as presented in Fig 2. As depicted, changes in mortality rates loosely follow changes in the PM_2.5_ concentrations, which can support our parallel trend assumption, although a reverse trend was observed in 2010.

**Table 1:** The descriptive summary of population, income, SEIFA, mortality, PM_2.5_, and temperature in Queensland.

Characteristic	Brisbane	Other states	Queensland
**Population**	2,109,466	2,579,668	4,689,134
<65 age (%)	87.04	82.84	84.75
≥65 age (%)	12.96	17.16	15.25
**Census account**	119	330	449
**Weekly income/person (median)**	725.0	597.0	623.5
**SEIFA**	4,132	3,867	3,910
**Death counts per year for sex**			
Female (%)	3,324 (51.91)	4,130 (48.35)	7,453 (49.21)
Male (%)	3,080 (48.09)	4,611 (51.65)	7,692 (50.79)
**Death counts per year for age groups**			
<65 age (%)	1,066 (16.65)	1,670 (19.11)	2,736 (18.07)
≥65 age (%)	5,338 (83.35)	7,071 (80.89)	12,409 (81.93)
**Death counts for diseases**			
Non-accidental	93,188	124,322	217,510
Cardiovascular	57,099	76,562	133,661
Respiratory	13,384	17,364	30,748
**Death counts per year**	102,464	139,856	242,320
**Environmental data**			
PM_2.5_ (median)	6.0 [5.0, 7.0]	3.0 [3.0, 3.8]	3.6 [3.0, 5.2]
Mean winter temperature	15.6 (2.0)	15.8 (2.4)	15.8 (2.7)
Mean summer temperature	24.8 (1.9)	25.9 (1.9)	25.6 (2.0)

Data are presented as mean (SD) for continuous normally distributed variables, median [IQR] for continuous non-normally distributed variables, or number (%) for categorical variables. Population, census account, and SEIFA are based on census 2016 data.

Abbreviations: IQR, interquartile range; PM_2.5_, fine particulate matter (particulate matter with a diameter of <2.5 μm); SD, standard deviation; SEIFA, Socio-Economic Indexes for Areas

In this study, we found a significant association between long-term exposure to PM_2.5_ and total mortality, with 2.02% (95% CI 1.41%–2.63%; *p* < 0.01) and 5.65% (95% CI 4.08–7.25%; *p* < 0.01) increases in total mortality per 1 μg/m^3^ increase in annual PM_2.5_ in Queensland and Brisbane, respectively. We also observed increases in cause-specific mortality associated with elevated PM_2.5_ levels **(**Table 2**)**. Higher effect estimates were observed in Brisbane than those in Queensland for all types of mortality.

**Table 2:** Associations between long-term PM_2.5_ and cause-specific mortality.

Mortality types	Brisbane		Queensland	
	Percent increase (95% CI)	*p*-Value	Percent increase (95% CI)	*p*-Value
**Non-accidental**	5.65 (4.08–7.25)	<0.01	1.92 (1.36–2.63)	<0.01
**Cardiovascular**	4.08 (2.02–6.18)	<0.01	1.41 (0.60–2.22)	<0.01
**Respiratory**	7.25 (3.05–11.29)	<0.01	5.44 (3.67–7.25)	<0.01
**Total**	5.65 (4.08–7.25)	<0.01	2.02 (1.41–2.63)	<0.01

Total mortality includes 7 kinds of classification of diseases (ICD-10: F00–F99, G00–G99, I00–I99, J00–J99, K00–K93, N00–N99, V01–Y98). Non-accidental causes include all the above diseases except for V01–Y98. Cardiovascular deaths (ICD-9: 390–459; ICD-10: I00–I99); respiratory causes (ICD-9: 460–519; ICD-10: J00–J99).

Abbreviation: PM_2.5_, fine particulate matter (particulate matter with a diameter of <2.5 μm)

We additionally investigated the association between long-term PM_2.5_ and cause-specific mortality grouped by age and sex **(**Table 3**)**. In Queensland, individuals under the age of 65 had a high risk of total, non-accidental, and cardiovascular death, with 5.76% (95% CI 4.29%–7.25%; *p* < 0.01), 6.18% (95% CI 4.29%–8.11%; *p* < 0.01), and 6.50% (95% CI 4.08%–9.20%; *p* < 0.01), respectively. In contrast, residents in Brisbane over the age of 65 were more likely to have elevated risk with 5.02% (95% CI 3.46%–6.50%; *p* < 0.01) and 4.08% (95% CI 2.22%–6.08%; *p* < 0.01) in non-accidental and cardiovascular mortality, respectively, for every 1 μg/m^3^ increase in annual average PM_2.5_. We also found larger effect estimates for the total mortality in females versus males in Queensland (2.84%, 95% CI 1.92%–3.67%; *p* < 0.01 versus 1.31%, 95% CI 0.50%–2.22%; *p* < 0.01).

**Fig 3:**
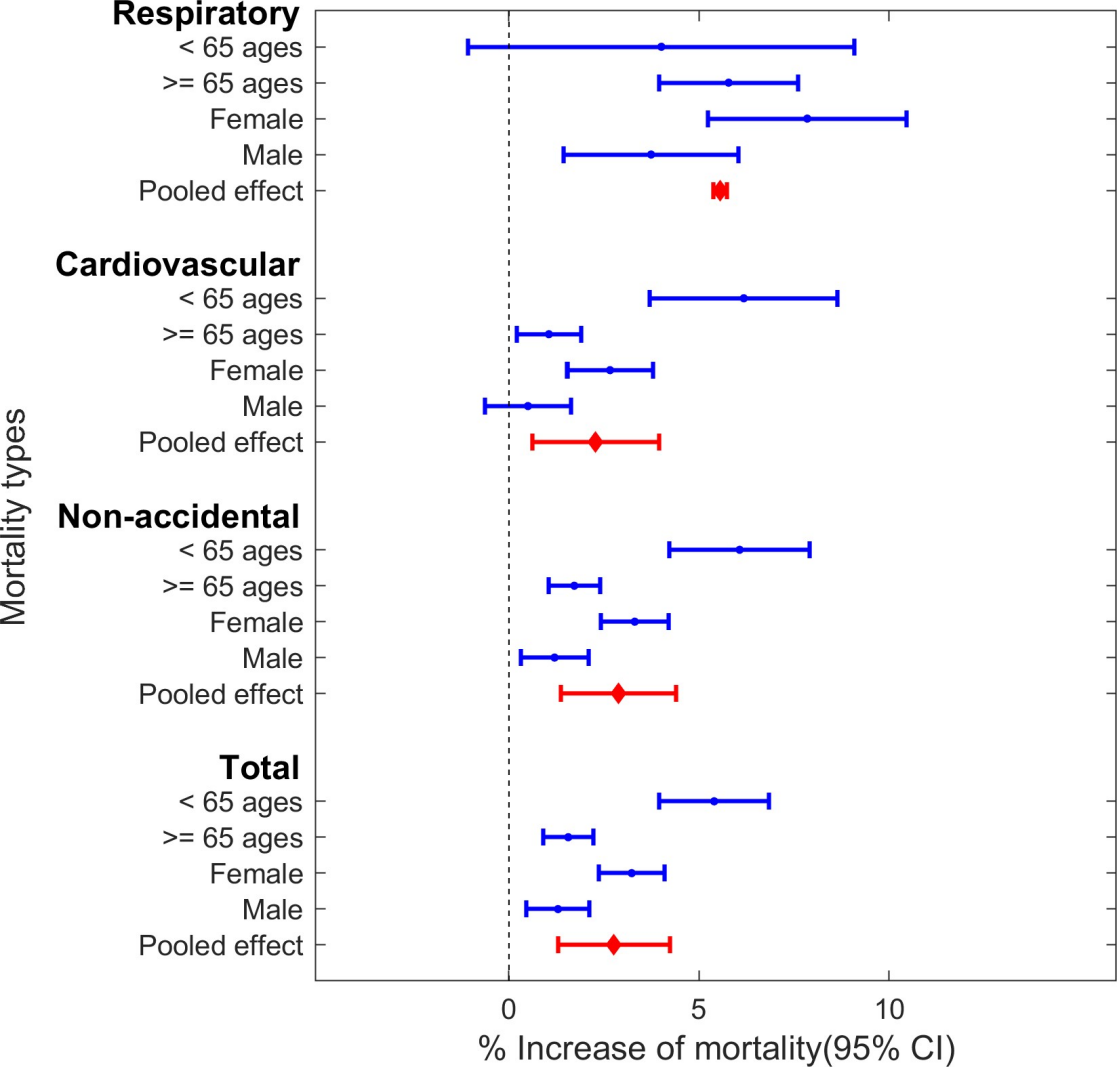
The pooled effects of PM_2.5_-mortality associations in Queensland by using a random effect meta-analysis. Total mortality includes 7 kinds of classification of diseases (ICD-10: F00–F99, G00–G99, I00–I99, J00–J99, K00–K93, N00–N99, V01–Y98). Non-accidental includes all above diseases except for V01–Y98. Cardiovascular (ICD-9: 390–459, ICD-10: I00–I99); respiratory causes (ICD-9: 460–519, ICD-10: J00–J99). PM_2.5_, fine particulate matter (particulate matter with a diameter of <2.5 μm).

**Fig 4:**
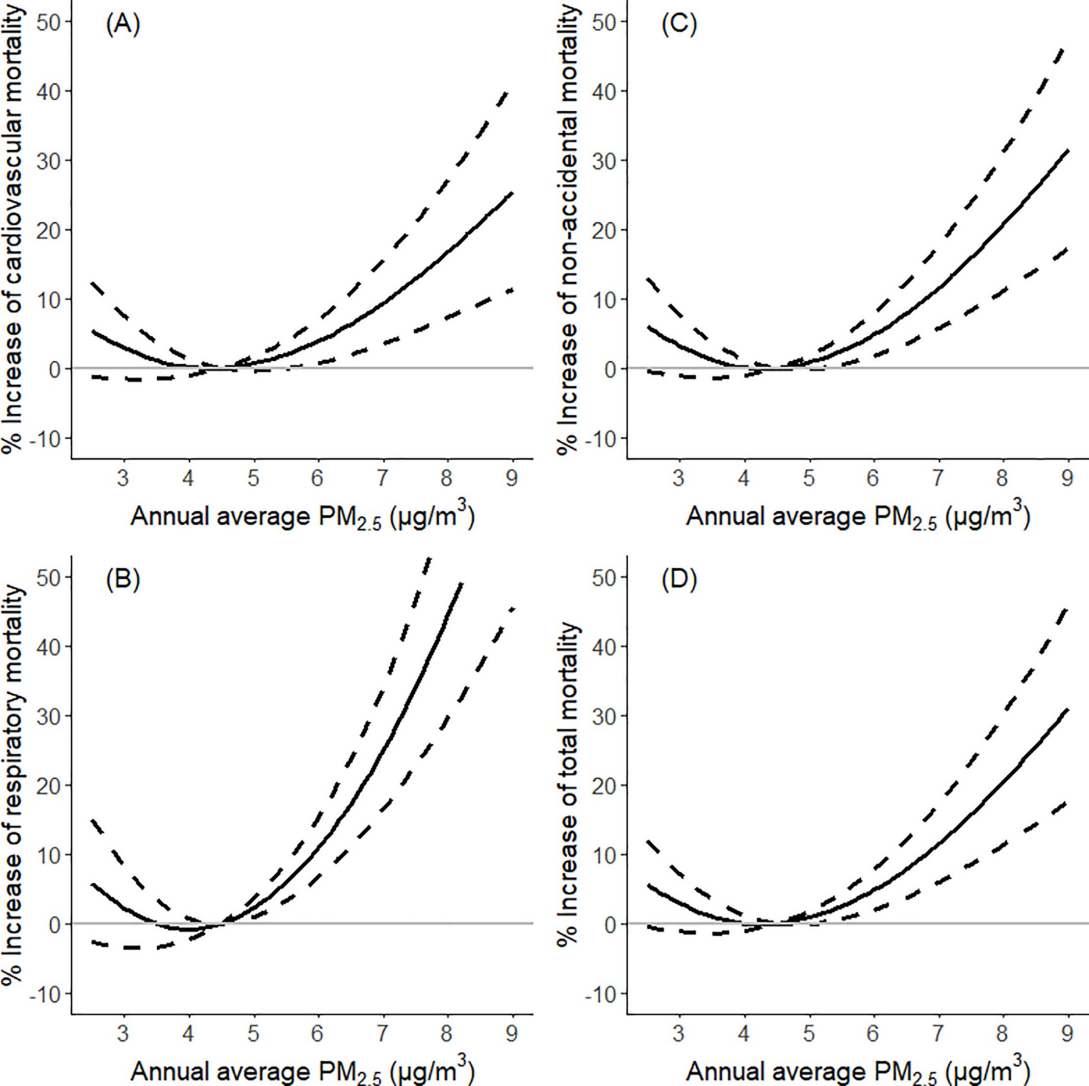
The association between annual average PM_2.5_ and the increase of cause-specific/total mortality in Queensland from 1998 to 2013. The association was examined using cubic splines with 3 degrees of freedom in generalized nonlinear models. Dotted lines: 95% CI; (A) cardiovascular causes: ICD-9: 390–459; ICD-10: I00–I99; (B) respiratory causes: ICD-9: 460–519; ICD-10: J00-J99; (C) non-accidental causes: ICD-10: F00–F99, G00–G99, I00–I99, J00–J99, K00–K93, N00–N99; and (D) total mortality: ICD-10: F00–F99, G00–G99, I00–I99, J00–J99, K00–K93, N00–N99, V01–Y98. PM_2.5_, fine particulate matter (particulate matter with a diameter of <2.5 μm).

Sensitivity analyses indicate our main findings are robust. With a random effect meta-analysis, we found that there were statistically significant pooled effects with a 2.76% (95% CI 0.61%–3.97%; *p* < 0.01) increase in total mortality, a 2.28% (95% CI 0.61%–3.97%; *p* < 0.01) increase in cardiovascular mortality, a 5.55% (95% CI 3.75%–7.39%; *p* < 0.01) increase in respiratory mortality, and a 2.88% (95% CI 1.37%–4.41%; *p* < 0.01) increase in non-accidental mortality (Fig 3 and S2 Table). The associations between PM_2.5_ and total/cause-specific mortality tend to be nonlinear, with a threshold around 4.5 μg/m^3^ for PM_2.5_ exposure (Fig 4). In addition, after modification by age group, we estimated a 5.21% (95% CI 3.20%–7.25%; *p* < 0.01) increase in total mortality among the <65 age group, whereas we estimated a 0.95% (95% CI −1.20% to 3.14%; *p* = 0.39) increase among subjects ≥65 years old, which were consistent with our model results (S3 Table). Moreover, the results were similar after adjusting for both the annual average and SD of temperature using natural splines with 3 and 4 degrees of freedom, respectively (S4 Table).

**Table 3:** Associations between long-term PM_2.5_ and cause-specific mortality in different ages and sexes in Queensland and Brisbane.

Subgroup	Area	Non-accidental		Cardiovascular		Respiratory		Total	
		Percent increase (95% CI)	*p*-Value	Percent increase (95% CI)	*p*-Value	Percent increase (95% CI)	*p*-Value	Percent increase (95% CI)	*p*-Value
<65 ages	Brisbane	2.33	0.26	−3.23	0.18	4.50	0.45	3.36	0.04
		(−1.59, 6.40)		(−8.39, 1.61)		(−6.76, 17.00)		(0.10, 6.61)	
	Queensland	6.18	<0.01	6.50	<0.01	3.98	0.13	5.76	<0.01
		(4.29, 8.11)		(4.08, 9.20)		(−1.19, 9.42)		(4.29, 7.25)	
≥65 ages	Brisbane	5.02	<0.01	4.08	<0.01	5.55	<0.01	4.92	<0.01
		(3.46, 6.50)		(2.22, 6.08)		(1.61, 9.75)		(3.46, 6.61)	
	Queensland	1.41	<0.01	0.70	0.07	5.65	<0.01	1.21	<0.01
		(0.70, 2.12)		(−0.10, 1.61)		(3.77, 7.57)		(0.60, 1.92)	
Female	Brisbane	7.79	<0.01	6.50	<0.01	11.29	<0.01	7.90	<0.01
		(5.76, 9.75)		(3.98, 9.09)		(5.55, 17.23)		(5.97, 9.86)	
	Queensland	2.84	<0.01	2.12	<0.01	7.57	<0.01	2.84	<0.01
		(1.92, 3.77)		(1.01, 3.36)		(4.92, 10.19)		(1.92, 3.67)	
Male	Brisbane	1.82	0.08	0.10	0.88	0.70	0.78	2.02	0.04
		(−0.20, 3.87)		(−2.37, 2.74)		(−4.21, 5.87)		(0.10, 3.87)	
	Queensland	1.21	<0.01	0.60	0.30	3.77	<0.01	1.31	<0.01
		(0.30, 2.02)		(−0.50, 1.82)		(1.41, 6.18)		(0.50, 2.22)	

Data are presented as the percent increase in death. Total mortality includes 7 kinds of classification of diseases mortality (ICD10: F00–F99, G00–G99, I00–I99, J00–J99, K00–K93, N00–N99, V01–Y98). Non-accidental causes include all the above diseases mortality except for V01–Y98. Cardiovascular deaths (ICD-9: 390–459; ICD-10: I00–I99); respiratory causes (ICD-9: 460–519, ICD-10: J00–J99).

Abbreviation: PM_2.5_, fine particulate matter (particulate matter with a diameter of <2.5 μm)

## Discussion

This study examined the association of low levels of PM_2.5_ (<9.0 μg/m^3^) with cause-specific mortality using a DID approach, which controls for potential unmeasured and omitted confounders. We found that long-term exposure to PM_2.5_ was associated with increased risks of total mortality and cause-specific mortality, despite low-level concentrations falling consistently below the current WHO annual standard (10 μg/m^3^). Furthermore, the effect estimates were higher in the urban area (Brisbane) when compared to statewide estimates for the study period.

In the presence of unmeasured confounders, it is difficult to determine causality in observational studies, especially for relatively weak health risk factors such as particulate matter. The PM_2.5_-mortality association may be affected by factors that are not perfectly measured or routinely collected, such as SES and outdoor physical activities. Therefore, if these unmeasured factors are omitted or unavailable, it might result in biased effect estimates [25,26]. It should be noted that many factors affect PM_2.5_-attributable mortality rates [27]. In addition to the variations witnessed in nonlinear exposure–response relationship across different causes of deaths [20,27–29], regional variations in ambient PM_2.5_, population characteristics, and baseline disease incidence rates all contribute to variations of PM_2.5_-attributable mortality [27]. Even though controlled exposure studies [30] and randomized participants [31] could reduce certain sources of bias and control for the unmeasured confounders, ethical considerations could be given on account of the potential for toxic components in the chemical composition of PM_2.5_.

However, a well-designed observational study such as a DID method may overcome the limitations of a nonrandomized observational study [18,32]. A case in a DID study is compared to itself at different time points so that certain confounders (including the unmeasured ones) such as population structure and lifestyle factors that remain stable or rarely varied during the study period are cancelled out because the comparisons occur among the population in the same places [15,19,33,34]. In our case, we controlled for certain slow-changing spatial-temporal variations by design, such as population and SES, which play a role in confounding the PM_2.5_-mortality association [18]. We also included seasonal temperature, which varies in different periods across the study areas and correlated to PM_2.5_ exposure and mortality as well as other potential factors. Controlling for temperature may indirectly remove the impacts of other factors like influenza epidemics. Therefore, we assumed that most unmeasured potential and omitted confounders had been controlled.

Several studies have used a similar DID design to investigate the potential association between long-term PM exposure and mortality [2,16–19]. For comparison purposes, we have converted the percent change in our study to a 10 μg/m^3^ increase and found a 22.14% (95% CI 15.02%–29.69%) increase in total mortality for the entire population of Queensland. This finding was slightly higher than that of Wang and colleagues [2], who developed a variant of DID approach to study 1938 census tracts in New Jersey from 2004 to 2009. They reported that each 10 μg/m^3^ increase in annual PM_2.5_ was associated with a 15.5% (95% CI 0.8%–32.3%) increase in the natural-cause mortality. Different outcome metrics might explain differences in results, e.g., we used 7 causes of death in Queensland instead of the natural-cause mortality. Another study estimated the city-specific health effects of PM_2.5_ on mortality in 207 US cities between 2000 and 2010. They observed a link between long-term PM_2.5_ and mortality with a hazard ratio (HR) of 1.2 (95% CI 1.1–1.3) for each 10 μg/m^3^ increase in annual PM_2.5_. However, direct comparisons between these results and the current study should be cautious because they combined the city-specific exposure, which tends to have different mixture composition of ambient particles [35].

The yields in this study were higher compared to many previous cohort studies [36–40]. For example, a recent representative cohort of American adults [40] estimated the HR of 1.12 (95% CI 1.08–1.15) for all-cause mortality and 1.23 (95% CI 1.17–1.29) for cardiopulmonary mortality per 10 μg/m^3^ long-term exposure to PM_2.5_. Most of the cohort studies are based on the exposure levels at a fixed time or area and consider some potential confounders that have been measured or observed. Our study considers the variability in exposure and potential influences in time and space, and the unmeasured confounders were captured by design. While cohort studies primarily rely on survey design and methodologies to obtain individual-level data, our study focuses on the spatiotemporal effects of PM_2.5_ on mortality at a macro scale by comparing differences in mortality and exposure of PM_2.5_ by postal code. In a systematic review, Vodonos and colleagues observed higher estimates in the hybrid space-time model than other methods [20]. In addition, another possible explanation for the relative high results in our study might be that the PM_2.5_ concentrations were much lower than in other studies, with a mean exposure of 3.63 μg/m^3^ (<9.00 μg/m^3^) in Queensland. Previous studies [20,41] have provided evidence for a nonlinear PM_2.5_-mortality association, where mortality increased sharply with low exposure levels and leveled off at higher exposure. A recent systematic review [20] estimated a 1.29% (95% CI 1.09–1.50) increase in all-cause mortality per 1 μg/m^3^ increase in PM_2.5_ at a mean exposure of 10 μg/m^3^, which decreased to 1.03% (95% CI 0.97–1.11) at 15.7 μg/m^3^ and to 0.82% (95% CI 0.52–1.12) at 30 μg/m^3^. They also restricted concentrations of PM_2.5_ to <10 μg/m^3^ and found a 2.4% (95% CI 0.80–4.00) increase per 1 μg/m^3^, which is consistent with our results.

Our results show a PM_2.5_-mortality association at levels below the current WHO air quality standard. Throughout the study period, the annual PM_2.5_ concentrations across Queensland areas were well below the current the WHO standard (10 μg/m^3^ of annual average PM_2.5_) and the US Environmental Protection Agency (EPA) standard (12 μg/m^3^ of annual average PM_2.5_); however, the PM_2.5_-mortality association remained present in spite of this. A growing body of research has suggested that low-level PM_2.5_ exposure may increase mortality. Shi and colleagues estimated a 9.28% increase in mortality for every 10-μg/m^3^ increase in annual PM_2.5_ for populations ≥65 years in the US. [42]. Aligning with our study, Markar and colleagues [3] and Schwartz and colleagues [4] used different causal reference methods to estimate the effects on mortality at concentrations of PM_2.5_ below the standards.

Mounting toxicological literature has provided evidence for the causal effect of PM_2.5_ on mortality [43–46]. For example, one study on animals [45] reported that mice with 6 months exposure to a low concentration of PM_2.5_ compared with animals exposed to filtered air demonstrated marked increases in atherosclerotic plaque, macrophage infiltration, and vasoconstrictor responses in the aortic arch. Another study [43] found more severe lung dysfunction in mice exposed for 8 months to PM_2.5_ of 16.8 μg/m^3^ when compared to animals exposed to PM_2.5_ of 2.9 μg/m^3^.

Several underlying biological mechanisms have been investigated for the damaging effects of PM_2.5_ on organ systems, especially on the respiratory and cardiovascular system [47–50]. The first is the direct pathway. Ultrafine particles directly translocate into the bloodstream and into specific organs, which aggravate the local oxidative stress and inflammation, causing the atherosclerotic plaque instability, and ultimately induce the cardiotoxicity effects and increase the risk of congestive heart failure, arrhythmias, and cardiovascular mortality [47–49]. Another pathway may increase oxidative stress and activate inflammation. The free radicals and organic components of PM_2.5_ can generate a rich milieu of inflammatory mediators and induce free radical production to oxidize lung cells, which directly causes cell injury in the lungs [51] and may indirectly release into the blood or systemic circulation, leading to cardiovascular and pulmonary disease and even death [50,52].

We also found that PM_2.5_ had larger effects on mortality in the urban area (Brisbane) when compared to statewide. In our study, the elderly inhabitants in Brisbane displayed a higher increase in death relative to statewide rates (4.92%, 95% CI 3.46%–6.61%; *p* < 0.01, versus 1.21%, 95% CI 0.06%–1.92%; *p* < 0.01). One possible explanation is the consistently higher concentrations of PM_2.5_ found in Brisbane (with an annual average of 6.0 μg/m^3^) compared to Queensland (with an annual average of 3.63 μg/m^3^). Furthermore, chemical and physical particle compositional differences in rural and urban areas also show different health effects on mortality [35,53]. Increasing evidence suggests that observed rural-urban disparities in population density could result in differences in PM_2.5_-associated mortality rates [54]. In our study, compared with the rest of Queensland, approximately half of the overall population (44.9% based on Census 2016 data) resides in Brisbane, resulting in more widespread exposure to PM_2.5_ than in rural areas (S2 Fig and S3 Fig).

Growing evidence has shown that the elderly are at a higher risk of total mortality attributable to particulate matter [42,55]. Contrary to these findings, however, our study found that young people (below the age of 65) in Queensland experienced higher risks comparatively (5.76%, 95% CI 4.29%–7.25%; *p* < 0.01, versus 1.21%, 95% CI 0.06%–1.92%; *p* < 0.01). In Brisbane, those over 65 had a higher risk of mortality associated with PM_2.5_ in comparison with people below 65. This may be explained by differential exposure and physical activity between urban and rural residents, which may vary by age [56]. Compared with older adults, young people in rural areas may have higher rates of outdoor physical activity, which may lead to greater air pollution exposure [57]. With regards to sex sub-analysis, in our study, females in Brisbane experienced a greater risk than females in Queensland. We found that females in our study had higher PM_2.5_-attributable mortality than the male population in both Brisbane and statewide. Even though an inverse relationship between all-cause mortality and PM_2.5_ exposure among female farmers was found in an American study [58], it is noted that there is a significant difference in individual-level behaviors between urban and rural residents, such as physical activity and smoking [59].

Our study has several strengths. To the best of our knowledge, no other studies have comprehensively explored the link between long-term exposure to PM_2.5_ and cause-specific mortality in Queensland when controlling for unmeasured confounding by design. Our study is based on 7 categories of disease-specific mortality, with a relevantly long study period (16 years). Additionally, we employed a variant of DID approach to explore the association between long-term exposure and mortality. Our results have provided compelling evidence for an association between long-term exposure to PM_2.5_ and mortality at levels below the current WHO air quality standard (10 μg/m^3^ of annual average PM_2.5_). Finally, we investigated the PM_2.5_-attributable mortality in the highly populated metropolis and found more severe risks on cause-specific mortality in Brisbane.

There are also some limitations to this study. We assumed that no predictors other than seasonal temperature exhibit different spatial-temporal variations in relation to PM_2.5_ exposure. However, there are other potential spatial and temporal confounders that vary by periods across the study areas and correlate with the PM_2.5_ exposure, such as employment rate [2,60] and influenza epidemic [15]. Furthermore, even though the DID study design may eliminate most unmeasured confounders, these factors held as ideal assume that confounders such as population, SEIFA, and some other unmeasurable factors like behavior habits in one area maintained unchanged over the study period. Additionally, our study was unable to measure individual-level exposure and potentially introduced ecological bias because the study was conducted on a population-level scale. Because the assessment of the environmental exposures was based on individual zip code, errors in geocoding and invariable yearly exposure in every postal code for all residents may lead to potential exposure misclassification. Moreover, we cannot estimate the association between PM_2.5_ and natural-cause mortality because of limited data availability. Furthermore, the basic population size and social-economic data in our study were based on Census 2016, which is likely to change over time.

This study provides evidence that long-term exposure to PM_2.5_, even at low levels well below the current WHO air quality standard, is associated with non-accidental, cardiovascular, and respiratory mortality in Queensland and Brisbane, by using a variant of DID approach to control the unmeasured confounding. Even though the explanation of the DID approach relies on several assumptions that are theoretical and, to some extent, unprovable, the findings are important for scientific understanding of the health effects of air pollution and to inform policy makers.

## Supporting information

S1 STROBE Checklist STROBE, Strengthening the Reporting of Observational Studies in Epidemiology. (DOC)

S1 Table Environmental and mortality data from 1998 to 2013. Temperatures are presented as mean (SD); registered deaths: the total registered death in Queensland; standardized death rate (‰) uses the registered death count (column 3/column 2) to divide the population. Study Death: the death count included in this study. PM_2.5_, fine particulate matter (particulate matter with a diameter of <2.5 μm); SD, standard deviation
(DOCX)

S2 Table The pooled effects of PM_2.5_-mortality associations for different death types by using a random effect meta-analysis. Total mortality includes 7 kinds of classification of diseases (ICD-10: F00–F99, G00–G99, I00–I99, J00–J99, K00–K93, N00–N99, V01–Y98). Non-accidental includes all above diseases except for V01–Y98. Cardiovascular (ICD-9: 390–459, ICD-10: I00–I99); respiratory causes (ICD-9: 460–519, ICD-10: J00–J99). PM_2.5_, fine particulate matter (particulate matter with a diameter of <2.5 μm)
(DOCX)

S3 Table The effect modification of PM_2.5_ on mortality by age group in Queensland and Brisbane. The effect modification by age groups was tested with the offset term of age-specific person-years. Data are presented as the percent increase in death (95% CI). Total mortality includes 7 kinds of classification of diseases mortality (ICD-10: F00–F99, G00–G99, I00–I99, J00–J99, K00–K93, N00–N99, V01–Y98). Non-accidental causes include all the above diseases mortality except for V01–Y98. Cardiovascular deaths (ICD-9: 390–459, ICD-10: I00–I99); respiratory causes (ICD-9: 460–519, ICD-10: J00–J99). PM_2.5_, fine particulate matter (particulate matter with a diameter of <2.5 μm)
(DOCX)

S4 Table Assessing the robustness of natural splines with different degrees for the summer and winter temperature in Queensland. Total mortality includes 7 kinds of classification of diseases (ICD-10: F00–F99, G00–G99, I00–I99, J00–J99, K00–K93, N00–N99, V01–Y98). Non-accidental includes all above diseases except for V01–Y98. Cardiovascular (ICD-9: 390–459, ICD-10: I00–I99); respiratory causes (ICD-9: 460–519, ICD-10: J00–J99). PM_2.5_, fine particulate matter (particulate matter with a diameter of <2.5 μm)
(DOCX)

S1 Fig The annual average PM_2.5_ concentrations in Brisbane during 1998–2013. PM_2.5_, fine particulate matter (particulate matter with a diameter of <2.5 μm). *The base map was obtained from Australian Statistical Geography Standard (ASGS), https://www.abs.gov.au/websitedbs/d3310114.nsf/home/digital+boundaries, CC BY 2*.*5 AU*.
(TIF)

S2 Fig The population density in Queensland based on 2016 census. *The base map was obtained from Australian Statistical Geography Standard (ASGS), https://www.abs.gov.au/websitedbs/d3310114.nsf/home/digital+boundaries, CC BY 2*.*5 AU*.
(TIF)

S3 Fig The population density in Brisbane based on 2016 census. *The base map was obtained from Australian Statistical Geography Standard (ASGS), https://www.abs.gov.au/websitedbs/d3310114.nsf/home/digital+boundaries, CC BY 2*.*5 AU*.
(TIF)

## Abbreviations

ASGS: Australian Statistical Geography Standard
DID: difference-in-differences
EPA: US Environmental Protection Agency
IQR: interquartile range
HR: hazard ratio
NAAQS: National Ambient Air Quality Standards
PM_2.5_: fine particulate matter
PM_10_: particulate matter with a diameter of less than 10 micrometers
SD: standard deviation
SEIFA: Socio-Economic Indexes for Areas
SES: socioeconomic status
SILO: Scientific Information for Land Owners
STROBE: Strengthening the Reporting of Observational Studies in Epidemiology
WHO: World Health Organization
