# Supplementary material for: The association between long-term exposure to low-level PM2.5 and mortality in the state of Queensland, Australia: A modelling study with the difference-in-differences approach
Source: PLoS Med. 2020 Jun 18;17(6):e1003141. doi: 10.1371/journal.pmed.1003141 (PMC7302440; doi:10.1371/journal.pmed.1003141)
Supplement: S1 Table — Temperatures are presented as mean (SD); registered deaths: the total registered death in Queensland; standardized death rate (‰) uses the registered death count (column 3/column 2) to divide the population. Study Death: the death count included in this study. PM2.5, fine particulate matter (particulate matter with a diameter of <2.5 μm); SD, standard deviation (DOCX) [file pmed.1003141.s002.docx]

**S1 Table.** Environmental and mortality data from 1998 to 2013

| Years | Population | Registered deaths | Standardized death rate | Study death | Average PM2.5 | | Winter temperature | Summer temperature |
| --- | --- | --- | --- | --- | --- | --- | --- | --- |
| 1998 | 3,404,484 | 22,321 | 6.56 | 14,327 | 3.31 | 16.38(2.59) | | 26.05(1.96) |
| 1999 | 3,453,936 | 22,849 | 6.62 | 14,526 | 3.86 | 15.65(2.08) | | 24.60(2.01) |
| 2000 | 3,509,458 | 22,425 | 6.39 | 14,244 | 3.74 | 15.08(2.08) | | 24.59(1.88) |
| 2001 | 3,571,469 | 22,856 | 6.40 | 14,296 | 4.00 | 15.84(2.01) | | 25.75(2.07) |
| 2002 | 3,653,123 | 23,968 | 6.56 | 14,925 | 3.71 | 15.44(2.30) | | 26.26(1.93) |
| 2003 | 3,743,121 | 23,500 | 6.28 | 14,513 | 3.29 | 16.08(2.49) | | 25.66(1.76) |
| 2004 | 3,829,970 | 24,514 | 6.40 | 15,257 | 3.37 | 15.63(2.41) | | 26.30(2.33) |
| 2005 | 3,918,494 | 23,584 | 6.02 | 14,572 | 3.57 | 16.24(2.14) | | 26.58(2.01) |
| 2006 | 4,007,992 | 24,473 | 6.11 | 15,285 | 3.12 | 15.93(1.89) | | 25.96(2.25) |
| 2007 | 4,111,018 | 25,801 | 6.28 | 15,925 | 3.03 | 15.08(2.69) | | 25.39(1.81) |
| 2008 | 4,219,505 | 27,335 | 6.48 | 13,938 | 3.00 | 15.48(2.65) | | 25.41(1.71) |
| 2009 | 4,328,771 | 26,316 | 6.08 | 14,922 | 5.28 | 16.44(2.88) | | 25.88(1.58) |
| 2010 | 4,404,744 | 27,289 | 6.20 | 15,782 | 2.01 | 16.03(2.10) | | 25.38(1.71) |
| 2011 | 4,476,778 | 27,414 | 6.12 | 16,601 | 3.31 | 15.03(2.06) | | 24.97(1.92) |
| 2012 | 4,568,687 | 28,300 | 6.19 | 16,784 | 4.00 | 15.28(2.36) | | 25.44(1.75) |
| 2013 | 4,652,824 | 27,901 | 6.00 | 16,423 | 3.29 | 16.44(2.24) | | 25.66(2.12) |

Temperatures are presented as mean (SD); registered deaths: the total registered death in Queensland; standardized death rate (‰) uses the registered death count (column 3/ column 2) to divide the population. Study Death: the death count included in this study. PM_2.5_, fine particulate matter (particulate matter with a diameter of < 2.5 µm); SD, standard deviation
