# Supplementary material for: The association between long-term exposure to low-level PM2.5 and mortality in the state of Queensland, Australia: A modelling study with the difference-in-differences approach
Source: PLoS Med. 2020 Jun 18;17(6):e1003141. doi: 10.1371/journal.pmed.1003141 (PMC7302440; doi:10.1371/journal.pmed.1003141)
Supplement: S2 Table — Total mortality includes 7 kinds of classification of diseases (ICD-10: F00–F99, G00–G99, I00–I99, J00–J99, K00–K93, N00–N99, V01–Y98). Non-accidental includes all above diseases except for V01–Y98. Cardiovascular (ICD-9: 390–459, ICD-10: I00–I99); respiratory causes (ICD-9: 460–519, ICD-10: J00–J99). PM2.5, fine particulate matter (particulate matter with a diameter of <2.5 μm) (DOCX) [file pmed.1003141.s003.docx]

**S2 Table.** The pooled effects of PM_2.5_-mortality associations for different death type by using a random effect meta-analysis.

| **Mortality type** | **% increase (95% CI)** | *P* value |
| --- | --- | --- |
| **Respiratory** |  |  |
| < 65 ages | 4.01(-1.07, 9.36) | 0.12 |
| ≥ 65 ages | 5.78(3.95,7.65) | <0.01 |
| Female | 7.85(5.24,10.52 | <0.01 |
| Male | 3.74(1.44,6.09) | <0.01 |
| Pooled effect | 5.55(3.75,7.39) | <0.01 |
| **Cardiovascular** |  |  |
| < 65 ages | 6.18(3.71,8.71) | <0.01 |
| ≥ 65 ages | 1.05(0.20,1.91) | 0.02 |
| Female | 2.66(1.53,3.81) | <0.01 |
| Male | 0.50(-0.64,1.66) | 0.39 |
| Pooled effect | 2.28(0.61,3.97) | <0.01 |
| **Non-accidental** |  |  |
| < 65 ages | 6.07(4.22,7.95) | <0.01 |
| ≥ 65 ages | 1.72(1.04,2.40) | <0.01 |
| Female | 3.31(2.41,4.21) | <0.01 |
| Male | 1.20(0.31,2.11) | <0.01 |
| Pooled effect | 2.88(1.37,4.41) | <0.01 |
| **Total** |  |  |
| < 65 ages | 5.40(3.95,6.87) | <0.01 |
| ≥ 65 ages | 1.56(0.89,2.22) | <0.01 |
| Female | 3.23(2.36,4.10) | <0.01 |
| Male | 1.29(0.46,2.14) | <0.01 |
| Pooled effect | 2.76(1.29,4.24) | <0.01 |

Total mortality includes 7 kinds of classification of diseases (ICD-10: F00–F99, G00–G99, I00–I99, J00–J99, K00–K93, N00–N99, V01–Y98). Non-accidental includes all above diseases except for V01–Y98.Cardiovascular (ICD-9: 390–459, ICD-10: I00–I99); respiratory causes (ICD-9: 460–519, ICD-10: J00–J99). PM_2.5_, fine particulate matter (particulate matter with a diameter of < 2.5 µm)
