# Supplementary material for: The association between long-term exposure to low-level PM2.5 and mortality in the state of Queensland, Australia: A modelling study with the difference-in-differences approach
Source: PLoS Med. 2020 Jun 18;17(6):e1003141. doi: 10.1371/journal.pmed.1003141 (PMC7302440; doi:10.1371/journal.pmed.1003141)
Supplement: S3 Table — The effect modification by age groups was tested with the offset term of age-specific person-years. Data are presented as the percent increase in death (95% CI). Total mortality includes 7 kinds of classification of diseases mortality (ICD-10: F00–F99, G00–G99, I00–I99, J00–J99, K00–K93, N00–N99, V01–Y98). Non-accidental causes include all the above diseases mortality except for V01–Y98. Cardiovascular deaths (ICD-9: 390–459, ICD-10: I00–I99); respiratory causes (ICD-9: 460–519, ICD-10: J00–J99). PM2.5, fine particulate matter (particulate matter with a diameter of <2.5 μm) (DOCX) [file pmed.1003141.s004.docx]

**S3 Table.** The effect modification of PM_2.5_ on mortality by age group in Queensland and Brisbane.

| Subgroup | Area | Non-accidental | | Cardiovascular | | Respiratory | | Total | |
| --- | --- | --- | --- | --- | --- | --- | --- | --- | --- |
|  |  | % increase (95% CI) | *P* -value | % increase | *P*-value | % increase | *P*-value | % increase | *P-*value |
|  |  |  |  | (95% CI) |  | (95% CI) |  | (95% CI) |  |
| < 65 ages | Brisbane | 3.06 | 0.22 | -3.08 | 0.28 | 5.77 | 0.35 | 3.86 | 0.05 |
|  |  | (-1.75, 8.11) |  | (-8.41, 2.56) |  | (-5.96, 18.97) |  | (-0.05, 7.92) |  |
|  | Queensland | 5.67 | <0.01 | 6.07 | <0.01 | 4.14 | 0.13 | 5.21 | <0.01 |
|  |  | (3.24, 8.17) |  | (3.00, 9.22) |  | (-1.35, 9.94) |  | (3.20, 7.25) |  |
| ≥ 65 ages | Brisbane | 14.27 | <0.01 | 13.9 | <0.01 | 14.1 | <0.01 | 14.23 | <0.01 |
|  |  | (8.97, 19.83) |  | (8.58, 19.47) |  | (7.08, 21.59) |  | (9.00, 19.72) |  |
|  | Queensland | 1.10 | 0.32 | 0.58 | 0.61 | 5.41 | <0.01 | 0.95 | 0.39 |
|  |  | (-1.07, 3.32) |  | (-1.64, 2.84) |  | (2.50, 8.39) |  | (-1.20, 3.14) |  |

The effect modification by age groups was tested with the offset term of age-specific person-years. Data are presented as the percent increase in death (95% CI). Total mortality includes 7 kinds of classification of diseases mortality (ICD-10: F00–F99, G00–G99, I00–I99, J00–J99, K00–K93, N00–N99, V01–Y98). Non-accidental causes include all the above diseases mortality except for V01–Y98.Cardiovascular deaths (ICD-9: 390–459, ICD-10: I00–I99); respiratory causes (ICD-9: 460–519, ICD-10: J00–J99). PM_2.5_, fine particulate matter (particulate matter with a diameter of < 2.5 µm)
