# Supplementary material for: The association between long-term exposure to low-level PM2.5 and mortality in the state of Queensland, Australia: A modelling study with the difference-in-differences approach
Source: PLoS Med. 2020 Jun 18;17(6):e1003141. doi: 10.1371/journal.pmed.1003141 (PMC7302440; doi:10.1371/journal.pmed.1003141)
Supplement: S4 Table — Total mortality includes 7 kinds of classification of diseases (ICD-10: F00–F99, G00–G99, I00–I99, J00–J99, K00–K93, N00–N99, V01–Y98). Non-accidental includes all above diseases except for V01–Y98. Cardiovascular (ICD-9: 390–459, ICD-10: I00–I99); respiratory causes (ICD-9: 460–519, ICD-10: J00–J99). PM2.5, fine particulate matter (particulate matter with a diameter of <2.5 μm) (DOCX) [file pmed.1003141.s005.docx]

**S4 Table.** Assessing the robustness of natural splines with different degrees for the summer and winter temperature in Queensland

| Cause-specific mortality | Degree = 3 | | | | Degrees = 4 | | | |
| --- | --- | --- | --- | --- | --- | --- | --- | --- |
|  | Percent Increase (%) | 95% CI | | *p-Value* | Percent Increase (%) | 95% CI | | *p-Value* |
| Non-accidental | 2.19 | 1.54 | 2.84 | <0.01 | 2.27 | 1.62 | 2.92 | <0.01 |
| Cardiovascular | 1.62 | 0.80 | 2.45 | <0.01 | 1.71 | 0.89 | 2.54 | <0.01 |
| Respiratory | 5.77 | 4.00 | 7.57 | <0.01 | 5.81 | 4.04 | 7.61 | <0.01 |
| Total | 2.19 | 1.58 | 2.81 | <0.01 | 2.27 | 1.65 | 2.89 | <0.01 |

Total mortality includes 7 kinds of classification of diseases (ICD-10: F00–F99, G00–G99, I00–I99, J00–J99, K00–K93, N00–N99, V01–Y98). Non-accidental includes all above diseases except for V01–Y98.Cardiovascular (ICD-9: 390–459, ICD-10: I00–I99); respiratory causes (ICD-9: 460–519, ICD-10: J00–J99). PM_2.5_, fine particulate matter (particulate matter with a diameter of < 2.5 µm)
