# Supplementary figures and images for: The association between long-term exposure to low-level PM2.5 and mortality in the state of Queensland, Australia: A modelling study with the difference-in-differences approach
Source: PLoS Med. 2020 Jun 18;17(6):e1003141. doi: 10.1371/journal.pmed.1003141 (PMC7302440; doi:10.1371/journal.pmed.1003141)

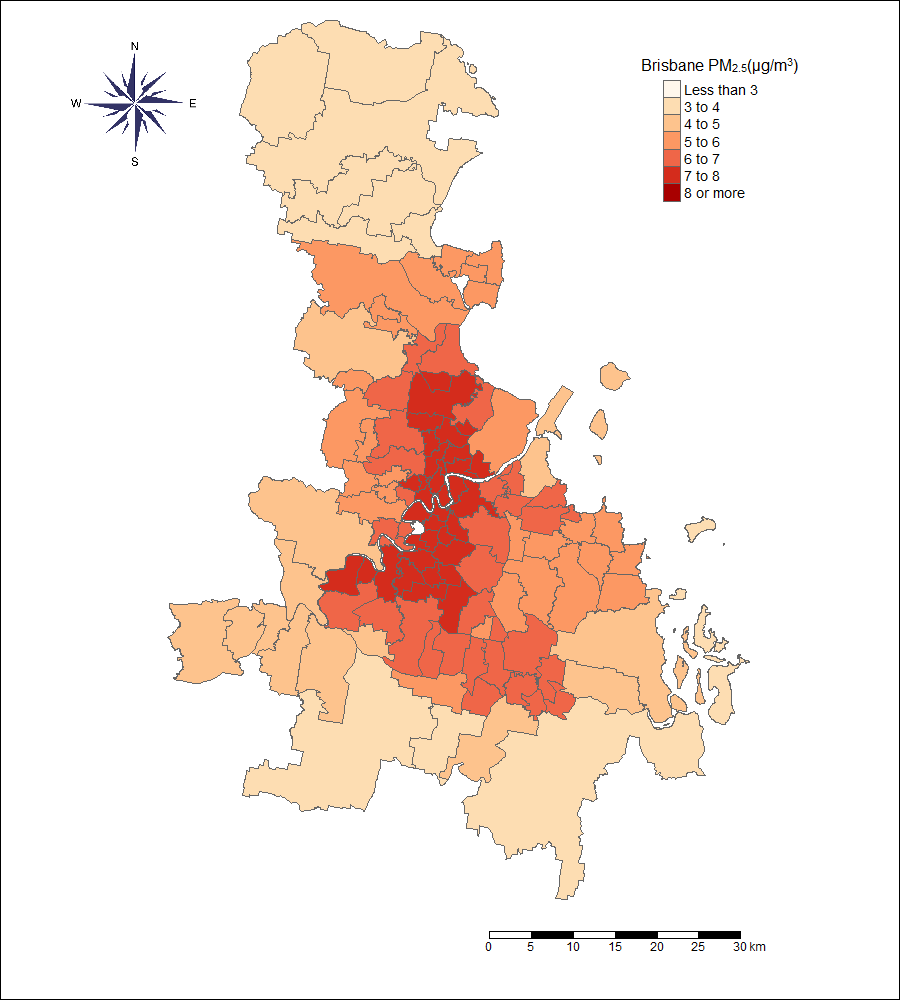

Supplement: S1 Fig — PM2.5, fine particulate matter (particulate matter with a diameter of <2.5 μm). The base map was obtained from Australian Statistical Geography Standard (ASGS), https://www.abs.gov.au/websitedbs/d3310114.nsf/home/digital+boundaries, CC BY 2.5 AU. (TIF) [file pmed.1003141.s006.tif]

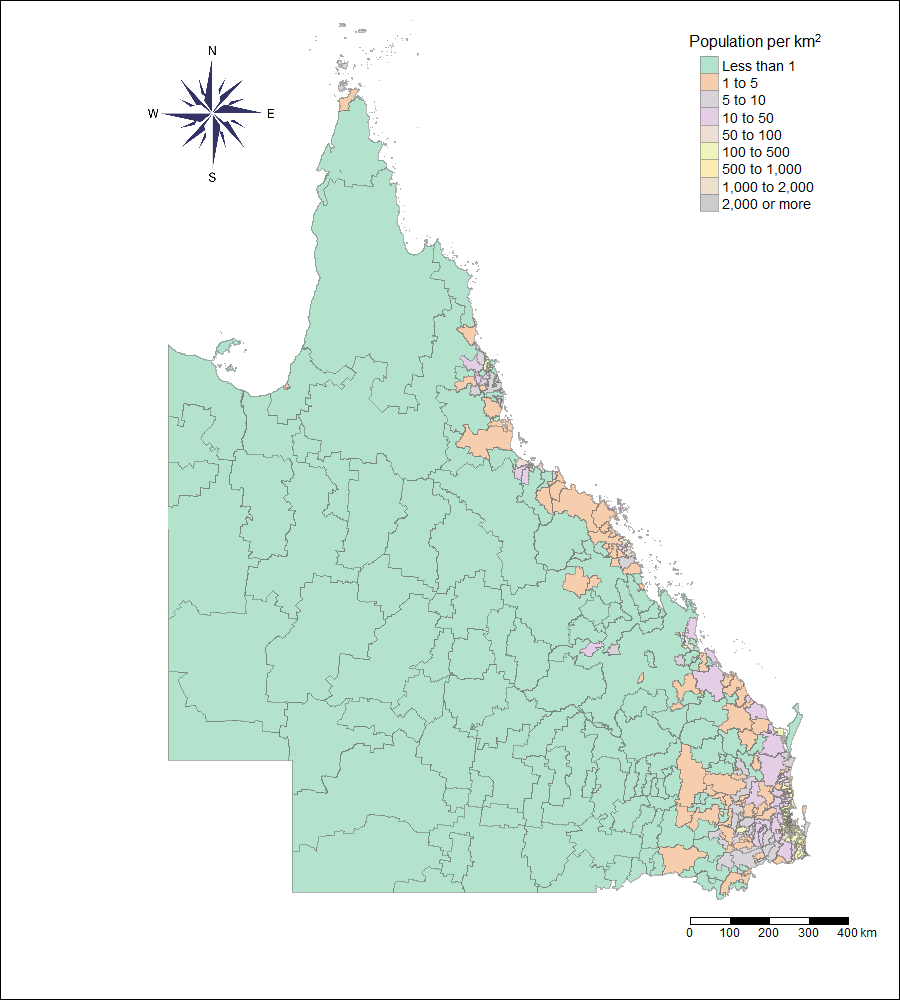

Supplement: S2 Fig — The base map was obtained from Australian Statistical Geography Standard (ASGS), https://www.abs.gov.au/websitedbs/d3310114.nsf/home/digital+boundaries, CC BY 2.5 AU. (TIF) [file pmed.1003141.s007.tif]

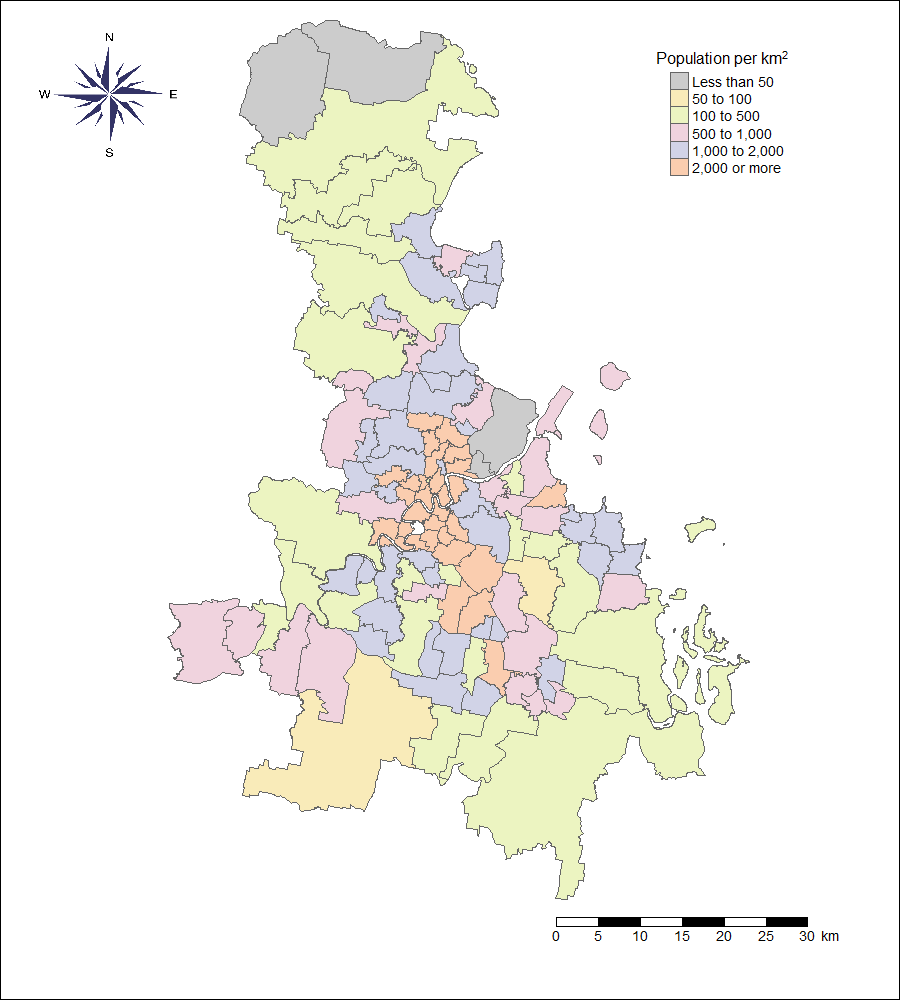

Supplement: S3 Fig — The base map was obtained from Australian Statistical Geography Standard (ASGS), https://www.abs.gov.au/websitedbs/d3310114.nsf/home/digital+boundaries, CC BY 2.5 AU. (TIF) [file pmed.1003141.s008.tif]
